# Reconsideration of importance of the point mutation L982W in the voltage-sensitive sodium channel of the pyrethroid resistant *Aedes aegypti *(L.)(Diptera: Culicidae) in Vietnam

**DOI:** 10.1371/journal.pone.0285883

**Published:** 2023-05-17

**Authors:** Hitoshi Kawada, Yukiko Higa, Shinji Kasai

**Affiliations:** 1 Institute of Tropical Medicine, Nagasaki University, Nagasaki, Nagasaki, Japan; 2 National Institute of Infectious Diseases, Shinjuku-ku, Tokyo, Japan; Institute of Zoology Chinese Academy of Sciences, CHINA

## Abstract

Pyrethroid resistance in *Aedes aegypti* is widespread in southern Vietnam because the photostable 2nd generation pyrethroids have been used in large amounts over extensive areas for malaria and dengue vector control. In our previous report in 2009, F1534C, one of the point mutations in the voltage-sensitive sodium channel (VSSC) in *Ae*. *aegypti*, was widespread at high frequency in south and central area. However, no significant correlation between the frequency of F1534C and pyrethroid susceptibility was detected primarily because the F1534C mutation frequency in the southern highland area was very low, despite that the bioassay indicated high pyrethroid resistance. The point mutation in the VSSC, L982W, which was not the target mutation in our previous study, was recently determined to be an important mutation causing high-pyrethroid resistance in Vietnamese *Ae*. *aegypti*. In the present study, a re-investigation of L982W in the mosquito samples collected in 2006–2008 revealed a greater distribution of this mutation (allelic percentage 59.2%) than F1534C (21.7%) and the greater proportion of homozygous L982W as compared to F1534C provided a plausible answer to the question concerning the unknown resistance factor in the southern highland area. L982W frequencies were uniformly higher in the southern part of Vietnam, including the highland area with a significantly high positive correlation with pyrethroid resistance in *Ae*. *aegypti*.

## Introduction

A pyrethroid is a general term for a group of structurally modified synthetic chemicals derived from the natural insecticidal products (pyrethrum) from the Dalmatian chrysanthemum, *Tanacetum cinerariifolium* (Trevir.) Sch. Bip. (1844), flowers. The pyrethroid group known as “knockdown agents,” or those categorized as “1st generation pyrethroids” possess a high knockdown activity but low killing activity. They have been used as a “spatial repellent” in anti-mosquito products, such as mosquito coils, mats, and vaporizer liquids. Low selection pressure by using such pyrethroids might result in the development of minimum physiological resistance in mosquito populations. However, the pyrethroids belonging to another group known as “killing agents” or those categorized as “2nd generation pyrethroids” generally exhibit high killing activity and photostability that enables outdoor use. The pyrethroids categorized in the latter group have been used as the predominant insecticides for vector control and agricultural pest control [1].

In Vietnam, photostable 2nd generation pyrethroids have been extensively used in large amounts for malaria and dengue vector control [2–5]. Consequently, several reports suggested the widespread distribution of pyrethroid resistance in *Aedes aegypti* (Linnaeus, 1762) in southern Vietnam [5–7]. Two different mutations in the voltage-sensitive sodium channel (VSSC), a predominant target of pyrethroids, F1534C and V1016G, were reported in Vietnamese *Ae*. *aegypti*. F1534C occurred at a relatively high frequency and was widespread, whereas V1016G was in limited areas at very low frequency [8]. Kawada *et al*. suggested the relationship between the F1534C and the amount of pyrethroids used in Vietnam but no significant correlation between the frequency of F1534C and pyrethroid susceptibility in *Ae*. *aegypti* was detected [8]. However, Kasai *et al*. recently reported that L982W, the point mutation first found and reported by Brengues *et al*. [9], which was not the target mutation in the previous report [8], plays an important role in pyrethroid resistance in Vietnamese *Ae*. *aegypti* [10].

The objective of the present study was to re-investigate mosquito samples collected during 2006–2008 in Vietnam [5, 8] to elucidate the importance of the point mutations in pyrethroid resistance in *Ae*. *aegypti* populations in Vietnam.

## Materials and methods

### Mosquitoes used in the study

#### Mosquitoes collected in the field survey during 2006–2008

**Fig 1:**
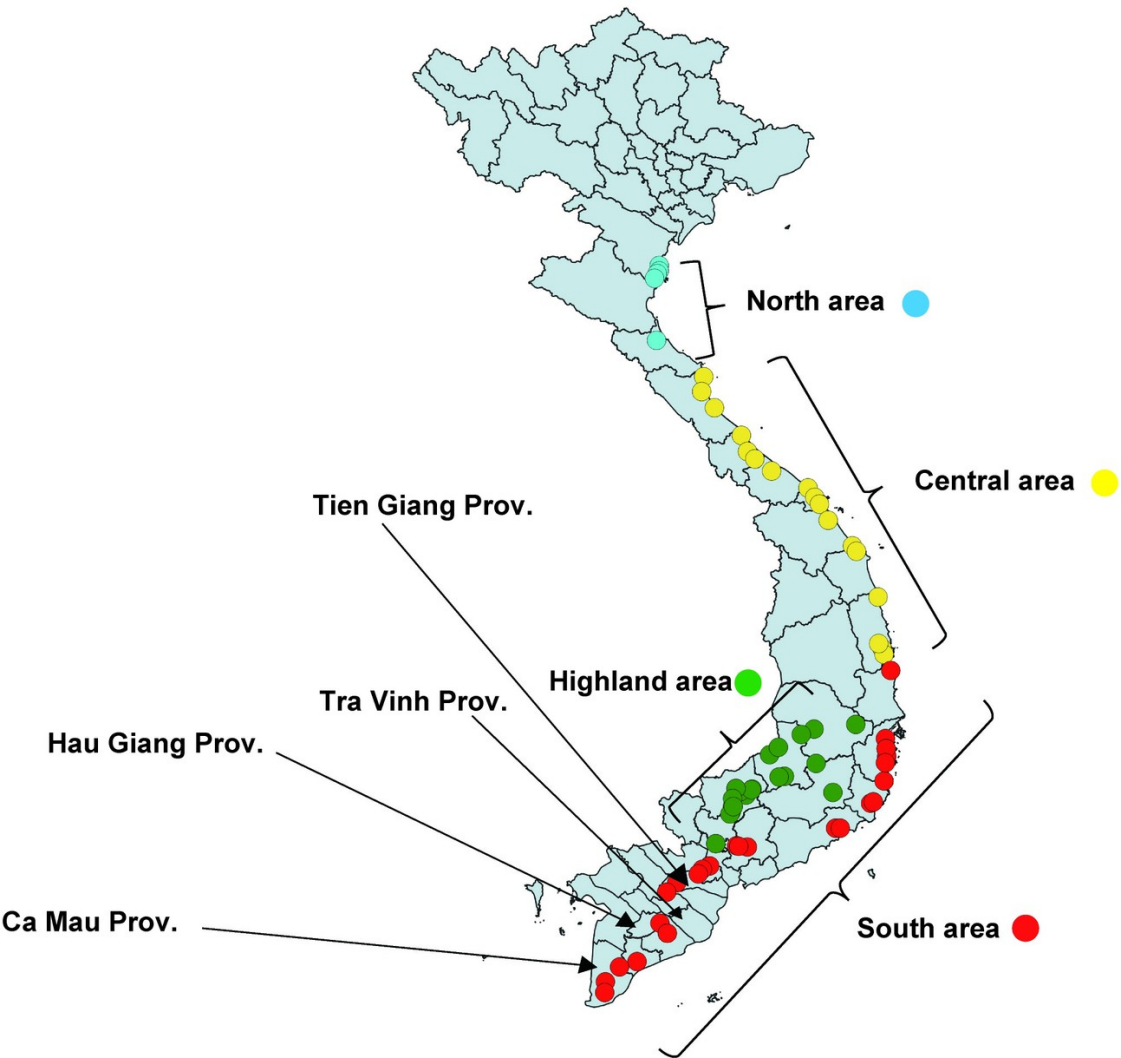
Map showing the mosquito sampling places (north, central, south, and highland area) in 2006–2008, and the provinces where the laboratory-reared *Ae*. *aegypti* colonies were collected. The shapefile was extracted from the GADM database (www.gadm.org).

Larval *Ae*. *aegypti* samples were collected from used tires along the national road in Vietnam (7–16 December 2006; 17–20 March, 15–20 May, and 1–12 July 2007; and 7–16 January 2008). According to geographical features, collection sites were categorized into four areas: north, central, highland, and south area as previously described by Kawada *et al*. [5] (Fig 1).

#### Laboratory mosquito colonies collected in southern Vietnam

Five laboratory-reared *Ae*. *aegypti* colonies collected in the Cai Lay District (10.4°N, 106.1°E), Tien Giang Province (TGCL colony), Chau Thanh A District (9.9°N, 105.6°E), Hau Giang Province (HGCT colony), Can Long District (10.0°N, 106.2°E), Tra Vinh Province (TVCL colony), Ca Mau Province (CMW colony), and the Cuu Nghia Community (10.4°N, 106.3°E), Tien Giang Province (TGTCN colony) during 2007–2008 were used (Fig 1). The mosquito colonies were transferred from the Pasteur Institute in Ho Chi Minh City. The MGK colony transferred from Sumitomo Chemical Co., Ltd., Hyogo, Japan which shows normal susceptibility to insecticides was used as a reference.

### Bioassay test method

#### Simplified knockdown bioassay using larvae

A simplified knockdown bioassay was conducted for the five laboratory-reared colonies (TGCL, HGCT, TVCL, CMW, and TGTCN), and one standard insecticide-susceptible colony (MGK colony transferred from Sumitomo Chemical Co., Ltd., Hyogo, Japan) were used in the bioassay according to the methods reported by Kawada *et al*. [5]. A mosquito larva was individually released in a glass vial containing 20 ml of water. A 250 ppm solution of *d*-T_80_-allethrin was obtained by diluting an 90% emulsifiable concentrate (formulated by Sumitomo Chem. Co., Ltd, Tokyo, Japan) with water, and 32 or 8 μl of the solution was added to the vial to obtain a concentration of 0.4 or 0.1 ppm, respectively. Twenty larvae from each colony were used for each concentration regime. Larval knockdown was observed for 30 min and the time to knockdown for each larva was recorded. The time required for knockdown was scored according to the six following categories: 1, <5 min; 2, 5–10 min; 3, 10–15 min; 4, 15–20 min; 5, 20–30 min; and 6, >30 min. A susceptibility index was calculated as the product of the scores at 0.1 and 0.4 ppm. Thus, mosquitoes with a susceptibility index = 1 were the most susceptible, and those with a susceptibility index = 36 were the least susceptible to *d*-allethrin.

The simplified knockdown bioassays for the mosquito samples collected in 2006–2008 were performed on the same day of mosquito collection. The bioassays for the laboratory-reared colonies (TGCL, HGCT, TVCL, CMW, and TGTCN) and MGK colony were performed in 2009.

#### Larval dipping test

A larval dipping test was conducted using the five laboratory-reared colonies (TGCL, HGCT, TVCL, CMW, and TGTCN) and one standard colony (MGK). An emulsifiable concentrate of 90% *d*-T_80_-allethrin was diluted with water to obtain a 0.1, 0.2, 0.4, and 0.8 ppm solution. In an additional dipping test, piperonylbutoxide (PBO) at a concentration of 0.6 ppm (for the TGCL, HGCT, TVCL, CMW, and TGTCN colonies) and 0.1 ppm (for the MGK colony) was mixed in each *d*-allethrin concentration for the detection of synergism. Ten larvae were released in 100 mL of the solution, and knockdown of the larvae was observed for 30 min to obtain the KT_50_ (time required for 50% knockdown). Mortality was observed 1 d after the test to obtain the LC_50_ (concentration required for 50% mortality). Two to four replicates were completed for each concentration.

#### Knockdown test using adult mosquitoes

Four laboratory-reared *Ae*. *aegypti* colonies (TGCL, HGCT, CMW, and TGTCN) and one standard colony (MGK) were used. A 0.5 g of mosquito coil piece was obtained by cutting the mosquito coils containing 0.3% *d*-T_80_-allethrin. A mosquito coil piece was completely burned in a glass chamber (70 × 70 × 70 cm). Immediately after the mosquito coil burned out, 20 3–5 d-old unfed female mosquitoes were released into the chamber, and their knockdown was observed for 20 min. Mortality was observed 1 d after the test. The test was performed in triplicate.

### Analysis of the point mutations in VSSC

The direct sequence was conducted to verify point mutations at L982, S989, V1016, and F1534 in *Ae*. *aegypti* samples. The whole body of a larva was placed in a 1.5 ml PCR reaction tube and homogenized in a mixed solution of extraction solution (20 μl) + tissue-preparation solution (5 μl) (REDExtract-N-Amp^TM^ Tissue PCR Kit; Sigma, St. Louis, MO, USA) for extraction of DNA. The solution was heated to 95°C for 3 min and was neutralized. Initial fragment amplification was conducted using primers AaSCF1 (AGACAATGTGGATCGCTTCC) and AaSCR4 (GGACGCAATCTGGCTTGTTA) for L982W, S989P, and V1016G analysis, and AaSCF7 (GAGAACTCGCCGATGAACTT) and AaSCR7 (GACGACGAAATCGAACAGGT) for F1534C analysis. The PCR mixture contained 4 μl of REDExtract-N-Amp^TM^ ReadyMix, 0.5 μM of each primer, and 1 μl of the DNA template for a total volume of 10 μl. PCR was performed under the following conditions: 94°C for 3 min and 35 cycles of 94°C for 15 s, 55°C for 30 s, 72°C for 30 s; and 72°C for 10 min. The amplified DNA were purified using ExoSAP-IT (USB Corporation, Cleveland, OH, USA) at a temperature of 37°C for 30 min and then at 80°C for 15 min. DNA sequencing was conducted using primers AaSCF3 (GTGGAACTTCACCGACTTCA) and AaSCR6 (CGACTTGATCCAGTTGGAGA) for L982W, S989P, and V1016G analysis and AaSCR8 (TAGCTTTCAGCGGCTTCTTC) for F1534C analysis. A BigDye Terminator v 3.1 Cycle Sequencing Kit (Applied Biosystems Japan, Ltd., Tokyo, Japan) was used for DNA sequencing according to the manufacturer’s instructions. Two micromoles of each primer were added to a tube with a total mixture volume of 10 μl. PCR was performed under the following conditions: 96°C for 1 min and 25 cycles at 96°C for 10 s, at 50°C for 5 s, and 60°C for 2 min. Ethanol precipitation for the samples was performed, and direct DNA sequencing was completed using the 3730 DNA Analyzer (Applied Biosystems). The electropherogram was analyzed by MEGA 4.0 public domain software (http://www.megasoftware.net/) and ATGC for Windows, version 9.0.0 (Genetyx Corporation, Tokyo, Japan).

The sequence data were obtained soon after the original collections (2006–2008) and were re-analyzed for the current study to calculate the frequency of L982W and S989P mutations. The unique DNA haplotype sequences were deposited in the GenBank database.

### Data analysis

The KT_50_ and LC_50_ were calculated according to the Bliss’ probit method [11]. Correlations between the susceptible index and arcsine converted allelic frequency of L982W and F1534C at each collection site of the mosquito samples collected in 2006–2008 (S1 Table) were analyzed using Pearson’s product-rate correlation coefficient using the software JMP 15.0 (SAS Institute Inc., Cary, NC, USA). Comparison of the collection number of homozygous and heterozygous point mutations, L982W and F1534C, in the north, central, highland, and south area in Vietnam was conducted with a χ^2^ test using JMP 15.0 software.

## Results

### Distribution of L982W in Vietnam, 2006–2008

**Fig 2:**
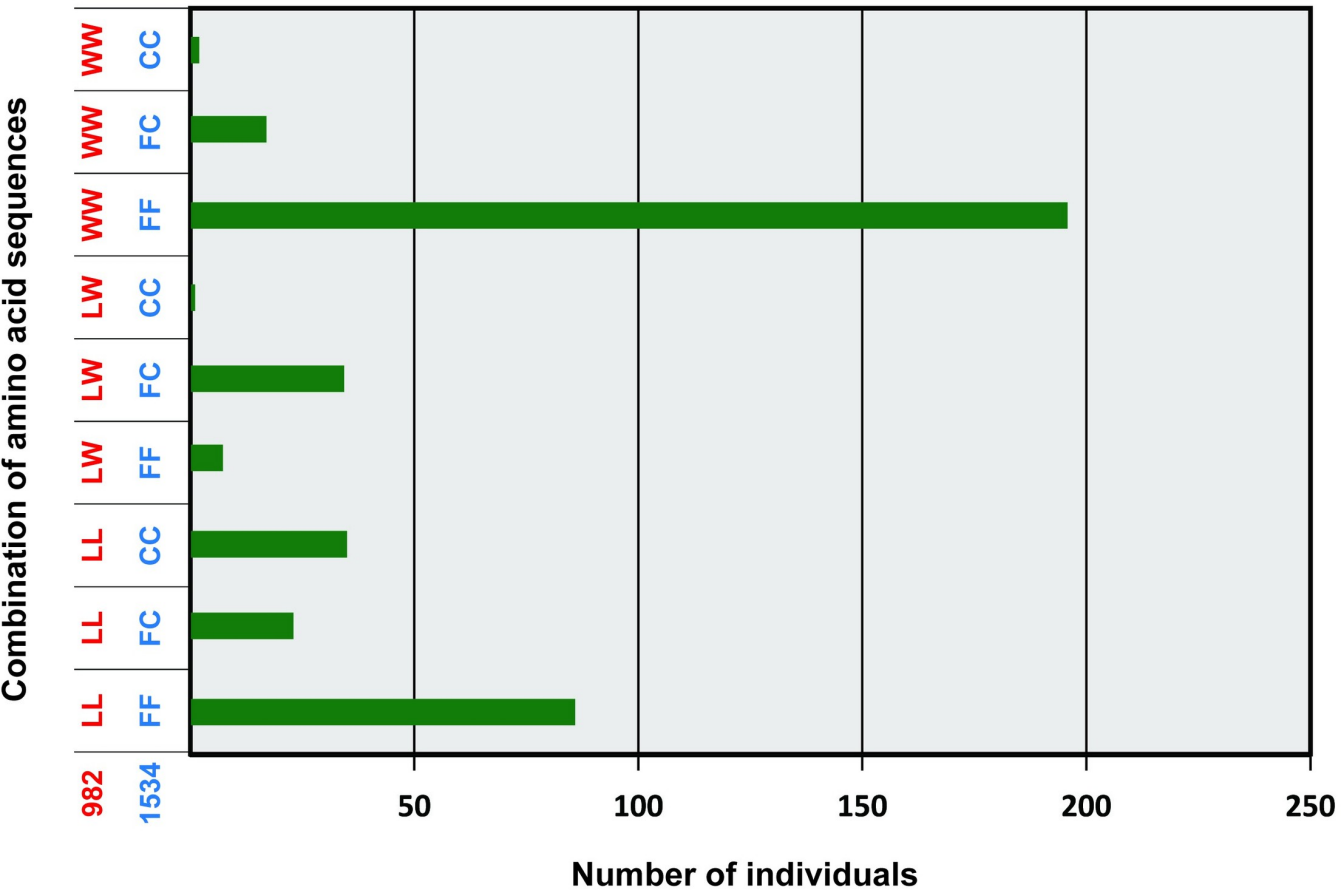
Genotyping of the L982W and F1534C point mutations in *Aedes aegypti* collected in Vietnam (2006–2008).

Among the 449 *Ae*. *aegypti* larvae collected from 68 locations, 240 (53.5%) and 50 (11.2%) larvae were homozygotic and heterozygotic for L982W, respectively (Accession LC639965). The overall percentage of 982W allele was 59.2% (S1 Table). Among 407 mosquito samples genotyped, the wild type samples which have neither L982W nor F1534C mutations (LL/FF) were 85 (20.9%). Co-occurrence of the two point mutations (L982W and F1534C) was detected in 58 mosquito samples among 407 samples (Fig 2). Among them, co-occurrence of heterozygotic L982W and heterozygotic F1534C (LW/FC) represented the majority (35 samples), followed by homozygotic L982W and heterozygotic F1534C (WW/FC, 17 samples). Co-occurrence of homozygotic L982W and homozygotic F1534C (WW/CC) samples was only detected in two mosquito samples (Fig 2). Co-occurrence of heterozygotic V1016G and heterozygotic F1534C (1 sample) were also detected. Additionally, only two S989P (Accession LC639964) heterozygotic individuals among 472 larvae from 68 locations were detected from one location, which was the Binh Son District, Quang Ngai Province, central east coast of Vietnam (site no. 1099) (S1 Table), and these two S989P mutations co-occurred with heterozygosis for V1016G [8] (Accession AB517741). When looking at the four loci (L982W, S989P, V1016G, and F1534C) simultaneously in 259 mosquito samples, the co-occurrence of heterozygotic S989P and V1016G did not co-occur with either L982W or F1534C (S2 Table).

**Fig 3:**
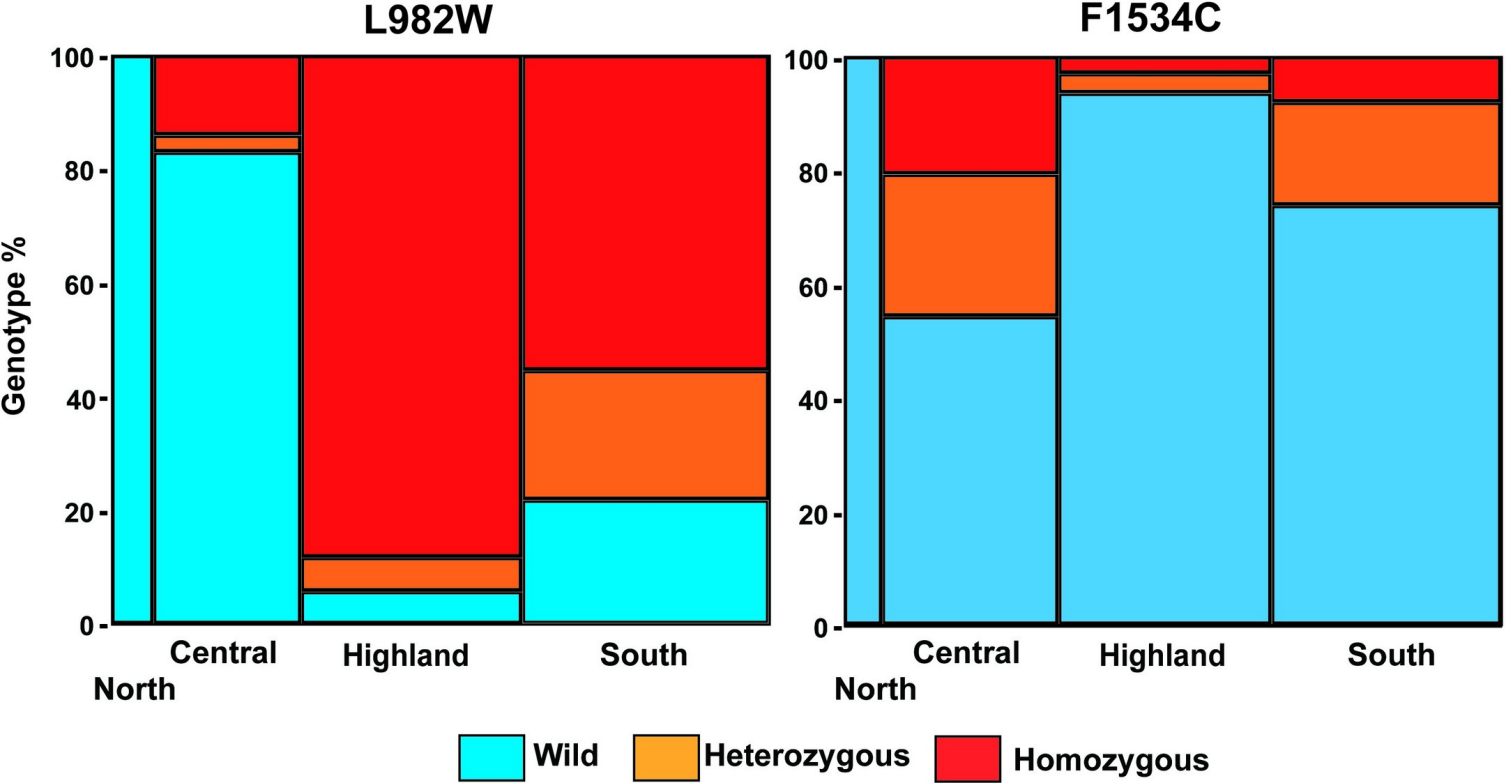
Mosaic diagram of the percentage of genotype for L982W and F1534C in the north, central, highland, and south area.

A significantly higher proportion of the homozygotic L982W mutation was distributed in the highland area of Vietnam (χ^2^ = 256, *p* < 0.0001), whereas a significantly higher proportion of homozygotic F1534C mutations were distributed in the central and south area compared to the highland area (χ^2^ = 65.2, *p* < 0.0001). The allelic L982W frequencies were found to be uniformly higher in the highland and south area of Vietnam as compared to the F1534C (Accession AB517740) frequencies (Tables 1 and 2 and Fig 3).

**Table 1:** Allelic frequency of the point mutations, L982W and S989P, in VSSC in *Aedes aegypti* collected in used tires in Vietnam.

Area	Mutation in VSSC											
	L982W						S989P					
	Total	W/W	W/L	L/L	Allelic %	Homozygotic %	Total	P/P	P/S	S/S	Allelic %	Homozygotic %
**North**	29	0	0	29	0	0	27	0	0	27	0	0
**Central**	101	14	3	84	15.3	13.9	124	0	2	122	0.81	0
**Highland**	151	133	9	9	91.1	88.1	151	0	0	151	0	0
**South**	168	93	38	37	66.7	55.4	170	0	0	170	0	0
**Total**	449	240	50	159	59.2	53.5	472	0	2	470	0.21	0

**Table 2:** Allelic frequency of the point mutations, F1534C and V1016G, in VSSC in *Aedes aegypti* collected in used tires in Vietnam.

Area	Mutation in VSSC^1^^)^											
	F1534C						V1016G					
	Total	C/C	C/F	F/F	Allelic %	Homozygotic %	Total	G/G	G/V	V/V	Allelic %	Homozygotic %
**North**	25	0	0	25	0.0	0	20	0	0	20	0	0
**Central**	116	24	29	63	38.1	20.7	86	0	2	84	0.81	0
**Highland**	140	4	5	131	4.3	2.9	99	0	0	99	0	0
**South**	150	12	27	111	15.2	8.0	104	0	0	104	0	0
**Total**	431	40	61	330	15.7	9.3	309	0	2	307	0.21	0

^1)^ Referred from Kawada et al. [8].

**Fig 4:**
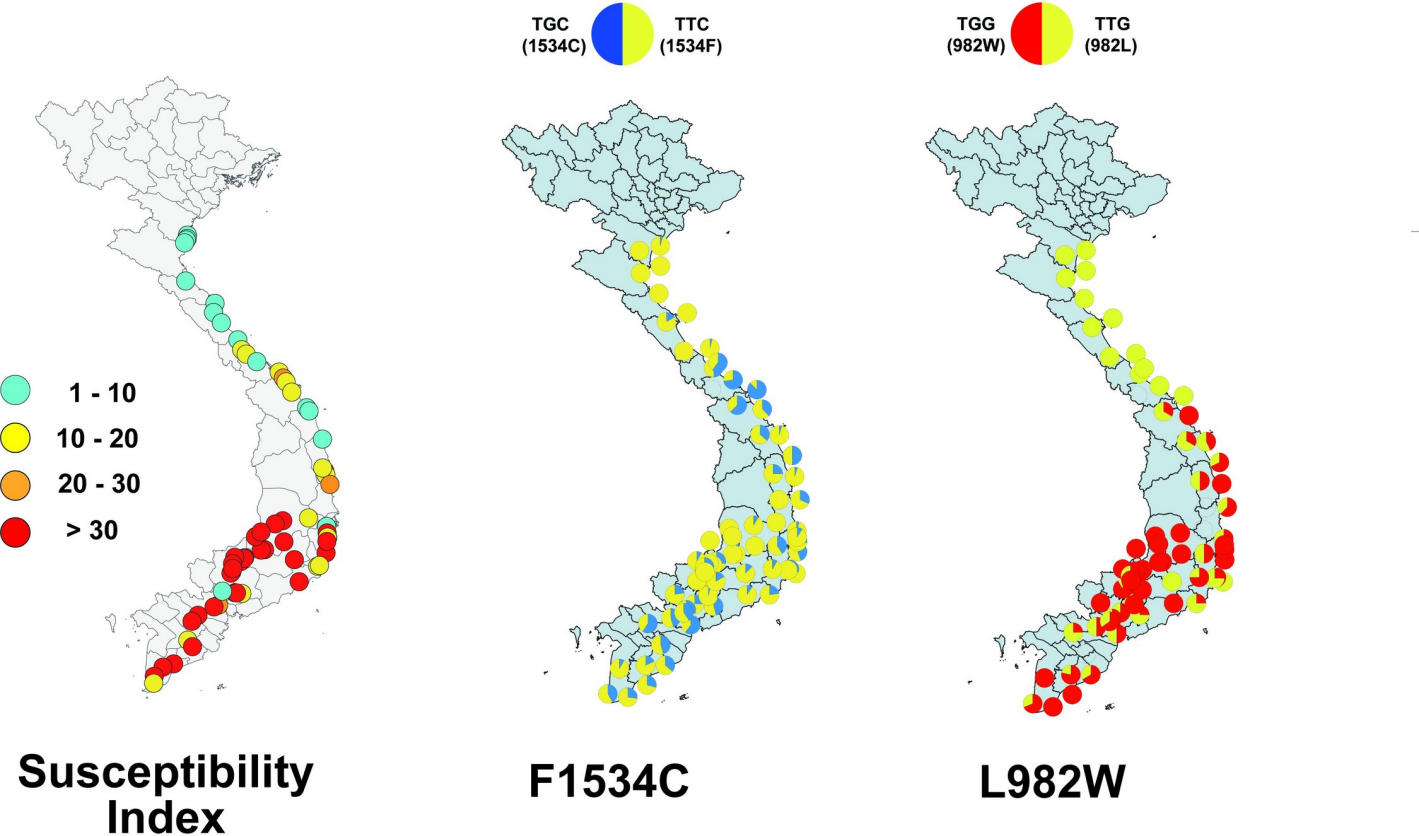
Distribution map of the point mutations in VSSC in *Ae*. *aegypti* collected in Vietnam. Circles in the left graph indicate the susceptibility indices based on a simplified knockdown bioassay. Pie charts in the middle and right graphs indicate the frequency of the point mutations, F1534C (modified from Kawada *et al*. [8]) and L982W. The shapefile was extracted from the GADM database (www.gadm.org).

The frequencies of F1534C appeared to be higher in the areas neighboring big cities, such as Dong Ha, Hue, Da Nang, Tam Ky, Quang Ngai, Quy Nhon, and Nha Trang [8]. In south and central area, high frequencies of F1534C were observed at almost all collection sites, except for the highland area (site nos. 3021, 3042, 3050, 3051, 5041, and 5042) located at an elevation of 500–800 m [8]. The allelic frequency of F1534C was very low (0–6.3%) in this area, although the susceptibility indices (calculated in the same manner as described in Materials and Methods) were at the maximum value 36, indicating high-pyrethroid resistance [8] (Fig 4).

**Fig 5:**
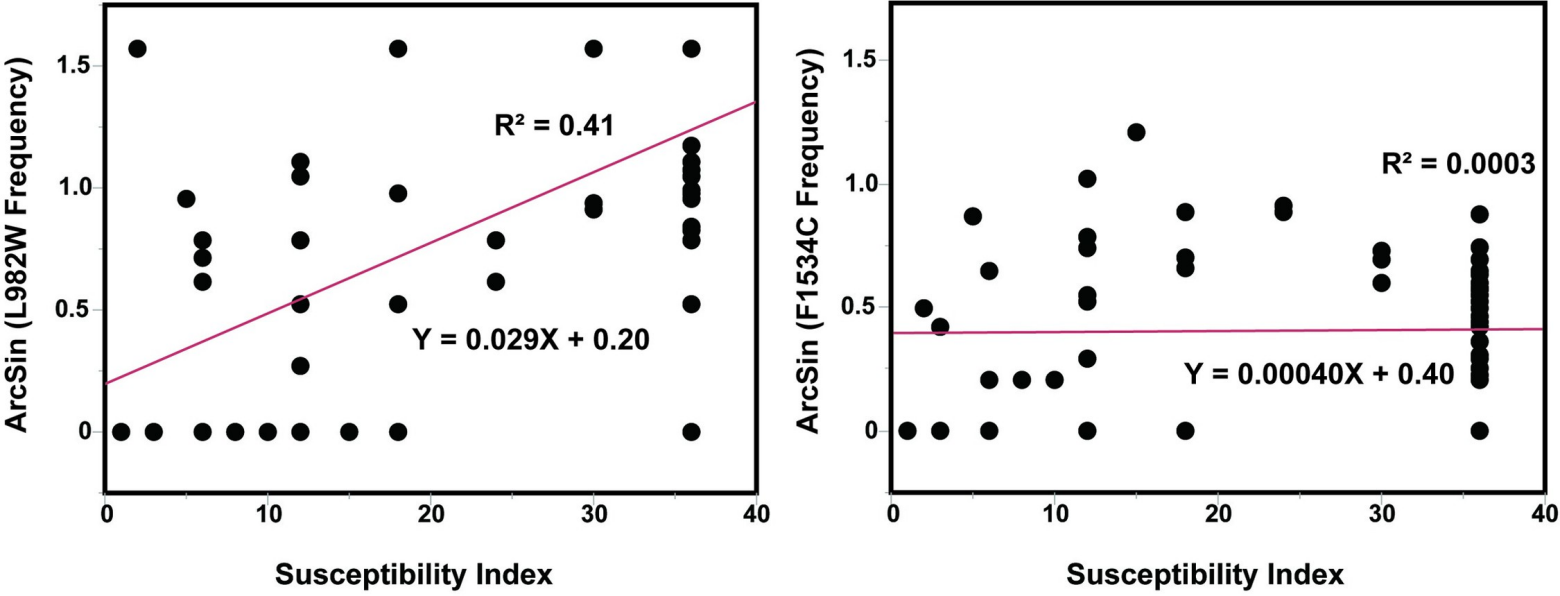
Correlation of susceptibility indices and the frequencies of L982W and F1534C at each collection site in Vietnam (2006–2008).

A significant positive correlation between the susceptibility indices and L982W frequencies was observed (R^2^ = 0.41, *p* < 0.0001), whereas no significant correlation was shown in F1534C (R^2^ = 0.0003, *p* = 0.89) (Fig 5). A negative correlation occurred between the L982W frequencies and latitude (R^2^ = 0.44, p < 0.0001) indicating that frequencies of L982W increased as the collection sites moved southward.

### Pyrethroid resistance in laboratory-reared *Ae*. *aegypti* colonies collected in southern Vietnam

The high allelic frequency of L982W was detected in the TGTCN (100%), CMW (50.0%), and TGCL colonies (20.8%). No S989P mutation was detected in the TGCL, HGCT, CMW, or TGTCN colonies. The V1016G mutation was detected in HGCT (21.3%), TGCL (4.3%), and TGTCN colonies (2.5%), whereas V1016G was not detected in TVCL or CMW colonies. F1534C was detected in the CMW (20.0%), TGTCN (16.1%), and TGCL colonies (13.2%). The F1534C mutation was not detected in the HGCT colony. No co-occurrence of plural mutations was detected, except for one individual in the TGTCN colony, which was homozygotic for both L982W and F1534C (Table 3).

**Table 3:** Larval and adult susceptibility against *d*-allethrin and frequency of point mutations in VSSC in the *Aedes aegypti* colonies collected in southern Vietnam.

Colony	Collection Site	Dipping test (Larvae)				Simplified knock down test (Larvae)	Knockdown test using mosquito-coil (Adult)	Allelic frequency (%)				Co-occurrence of homozygotes (%)		
		PBO (ppm)	Concentration to get KT_50_ ^1^^)^ = 20 min. (ppm)	LC_50_ ^2^^)^ (95% CI^3^^)^) (ppm)	Synergism of PBO	Susceptibility Index	KT_50_ (95% CI^3^^)^) (min.)	L982W	S989P	V1016G	F1534C	L982W+F1534C	S989P+V1016G	V1016G+F1534C
**TGCL**	**Cai Lay Dist., Tien Giang Prov.**	0	0.4–0.8	0.25 (0.20–0.34)	1.47	36	10.3 (9.38–11.4)	20.8	0	4.3	13.2	0	0	0
		0.6	> 0.8	0.17 (0.13–0.37)										
**HGCT**	**Chau Thanh A Dist., Hau Giang Prov.**	0	0.4–0.8	0.28 (0.24–0.34)	2.23	36	6.17 (5.78–6.60)	0	0	21.3	0	0	0	0
		0.6	0.4–0.8	0.13 (0.10–0.15)										
**TVCL**	**Can Long Dist., Tra Vinh Prov.**	0	> 0.8	0.25 (0.19–0.31)	1.71	36	NA ^4^^)^	NA	NA	0	NA	NA	NA	NA
		0.6	> 0.8	0.15 (0.12–0.18)										
**CMW**	**Ca Mau Prov.**	0	0.924	0.21 (0.12–0.34)	1.50	36	> 20	50.0	0	0	20.0	0	0	0
		0.6	0.745	0.15 (0.090–0.20)										
**TGTCN**	**Than Cuu Nghia Com., Tien Giang Prov.**	0	> 0.8	0.49 (0.40–0.66)	0.98	36	> 20	100	0	2.5	16.1	2.8	0	0
		0.6	> 0.8	0.44 (0.35–0.59)										
**MGK**	**-**	0	0.1–0.2	0.11 (0.086–0.20)	1.29	12	7.83 (7.37–8.34)	0	0	0	0	0	0	0
		0.1	0.1–0.2	0.082 (0.070–0.098)										

^1)^ KT_50_, time required for 50% knockdown,

^2)^ LC_50_, concentration required for 50% mortality,

^3)^ Confidence Interval,

^4)^ NA, not available.

The maximum susceptibility index 36 occurred in all colonies, indicating high-pyrethroid resistance in these colonies. The TGTCN colony exhibited the highest resistance to *d*-allethrin in the larval dipping test (LC_50_ = 0.490 ppm), followed by the HGCT (LC_50_ = 0.283 ppm), TVCL (LC_50_ = 0.252 ppm), TGCL (LC_50_ = 0.250 ppm), and CMW colonies (LC_50_ = 0.210 ppm). No synergism from the addition of PBO was shown in TGTCN (synergism 98) compared to that of other colonies (synergism 147–223). Synergism with PBO was the most prominent in the HGCT colony (synergism = 223), indicating high cytochrome P450 monooxygenase (P450) related metabolic factors in this colony. The frequency of L982W seems to play a significant role in the delayed knockdown time in adult mosquito bioassay (Table 3).

## Discussion

Previously, we revealed that the F1534C mutation was observed in almost all collection sites in the central and southern parts of Vietnam [8]. However, the frequencies of the F1534C mutation in the highland area were very low, and the possible existence of different resistance mechanisms, such as unknown novel point mutations and/or an enhanced detoxification pathway associated with pyrethroid insensitivity, has been suggested [8]. Re-investigation of the L982W mutation in the present study encouraged by the new finding of the importance of L982W in *Ae*. *aegypti* resistance by Kasai *et al*. [10] revealed a more frequent distribution of this point mutation than F1534C and provided a plausible answer to the above question concerning the unknown resistance factor in the highland area. Malaria and dengue hemorrhagic fever (DHF) are serious arthropod-borne diseases in Vietnam and other Asian countries. The incidence of malaria in Vietnam has declined dramatically from more than one million cases with 4,500 deaths in 1991 to approximately 10,000 cases and three deaths in 2015 [12, 13] because of an enhanced malaria control program and extensive socio-economic development [12]. However, malaria remains a problem in the highlands, despite control efforts including enhanced health services, free administration of artemisinin drugs, and free delivery of insecticide-treated nets [12]. A total of 7,710 malaria cases and drug-resistant *Plasmodium* parasites have been reported in the highland in 2014 [13]. Pyrethroid treatment for malaria control, such as indoor residual sprays (IRS) and insecticide-treated bed nets (ITNs), have been intensively used in the interior and periphery of human habitation areas. Incidentally, these were the breeding and resting sites of domestic and indoor *Ae*. *aegypti*, resulting in strong selection pressure. Additionally, 21,000 L of photostable pyrethroid formulations, such as λ-cyhalothrin, deltamethrin, and permethrin, were reportedly used for dengue control in 20 southern provinces in 2007 (Pasteur Institute, 2008). The decrease in pyrethroid susceptibility in *Ae*. *aegypti*, according to the decrease in the latitude of the collection points, have been reported in previous studies [6–8]. The above authors concluded that the extended use of pyrethroids (λ-cyhalothrin, α-cypermethrin, deltamethrin, and permethrin) for malaria control might be one factor that caused pyrethroid resistance in *Ae*. *aegypti*. Huong and Ngoc reported high resistance to λ-cyhalothrin, deltamethrin, permethrin, and DDT in eight *Ae*. *aegypti* colonies collected in the highland and concluded that the resistance was possibly caused by the extended use of these insecticides in malaria and dengue control [14].

Although we could not test the mosquito colony in the highland area, genotyping and susceptibility test using *d*-allethrin for the five colonies sampled in southern Vietnam revealed a high correlation between the frequency of L982W and pyrethroid resistance. L982W was first detected and reported by Brengues *et al*. [9] and the pyrethroid resistance in the permethrin-selected *Ae aegypti* colony (LHP) with L982W originating in Vietnam was reported by Pennetier *et al*. [15]. However, the importance of this point mutation in pyrethroid resistance in Vietnamese *Ae*. *aegypti* was not clarified until the report of Kasai *et al*. [10], who revealed that co-occurrence of the two point mutations, L982W and F1534C, created the highest resistance to permethrin (>1000 as compared to a susceptible colony). In contrast, the resistance ratio with a single mutation of L982W or F1534C resulted in 52 to 75-times and 8-times, respectively. The authors showed that the allelic frequency of L982W was >79% in the mosquito colonies collected in Hanoi, Dak Lak, and Ho Chi Minh. Their report and the present study indicate that the allelic frequency of L982W increased during this decade (2006–2016). Moreover, the co-occurrence of L982W and F1534C also appeared to increase. In fact, co-occurrence of the homozygous L982W and F1534C was only 0.49% (2/407) in the mosquito samples collected in our study (2006–2008), while the frequency of the double mutations (L982W+F1534C) in 2016 was estimated to be 28.4–40.5, 11.8–16.5, and 6.2–7.3% in the populations from Hanoi, Dak Lak, and Ho Chi Minh City, respectively [10]. Furthermore, it should be noted that not a single individual with wild haplotype (982L+1534F) was detected by Kasai et al. in 2016 [10], whereas 20.9% of the mosquito samples were with the wild haplotype in our study (2006–2008).

Co-occurrence of two or three point mutations appears to be a common phenomenon in pyrethroid-resistant *Ae*. *aegypti* populations [16]. V1016G was often associated with S989P in Southeast Asia [17–19]. Double mutations, V1016G and F1534C, and triple mutations, S989P, V1016G, and F1534C, were detected in southern China and Southeast Asian countries [20–24]. V1016I has been found to coexist with F1534C in South and North America [25–31] and Africa [32]. Among the above co-occurrence of point mutations in VSSC, the triple mutation with S989P, V1016G, and F1534C is thought to cause the greatest reduction (1,100-fold) in permethrin susceptibility in *Ae*. *aegypti* based on VSSC expression in *Xenopus* oocytes in an electrophysiological study [33]. However, the greater resistance ratio in the double mutation with L982W and F1534C compared to the triple mutation above was reported by Kasai *et al*. [10] using a bioassay with intact *Ae*. *aegypti* colonies expressing the mutation. Kasai *et al*. reported that L982W and the co-occurrence of L982W and F1534C with much higher frequencies than Vietnam were found in the *Ae*. *aegypti* colonies in Cambodia, which shares a border with southern Vietnam, indicating the possible introgression of these mutation genes between the two countries [10]. The importance of monitoring the occurrence of L982W and L982W + F1534C mutations, is therefore, strongly encouraged for the effective prevention of dengue outbreaks regardless of insecticidal intervention.

## Supporting information

S1 Table Susceptibility index and allelic frequency of the point mutations, L982W and S989P, in VSSC in *Aedes aegypti* collected in used tires in Vietnam. (XLSX)

S2 Table Distribution and co-occurrence of the point mutations, L982W, S989P, V1016G, and F534C in 259 samples of *Ae*. *aegypti* collected in Vietnam (2006–2008). (XLSX)
